# Panniculitis as the Transformed Cutaneous Manifestation of Refractory Dermatomyositis with Successful Management with Tofacitinib

**DOI:** 10.31138/mjr.33.3.380

**Published:** 2022-09-30

**Authors:** Upendra Rathore, Neha Nigam, Amita Aggarwal, Latika Gupta

**Affiliations:** 1 Department of Clinical Immunology and Rheumatology, Sanjay Gandhi Post Graduate Institute of Medical Sciences, Lucknow, India,; 2 Department of Pathology, Sanjay Gandhi Post Graduate Institute of Medical Sciences, Lucknow, India

A 26-year-old girl presented in 2017 with fever, proximal muscle weakness, and cutaneous rashes of one-year duration. A diagnosis of Dermatomyositis (DM) was made based on heliotrope and malar rashes, Gottron’s sign, and cutaneous ulcers on the elbows, and significant proximal muscle weakness with a manual muscle testing score of 37/80 with elevated muscle enzymes (AST- 85U/L, ALT-53U/L, LDH-701U/L, CPK-303U/L). Myositis specific antibodies (MSA) were negative. Upon initiating 1 mg/kg glucocorticoids and methotrexate, a rapid improvement in muscle weakness was recorded over 8 weeks though cutaneous disease persisted. Subsequently various drugs (Hydroxychloroquine, Rituximab, Tacrolimus, Mycophenolate mofetil and Thalidomide) were unsuccessfully tried for refractory cutaneous disease over the next two years. Eventually Intravenous Immunoglobulin (IvIg) (2gm/kg 4-weekly doses) was initiated, following which rashes subsided and glucocorticoids were tapered and stopped over the next ten months.

The ongoing pandemic led to disruption of infusions for three months due to a nationwide lockdown. The patient returned with painful subcutaneous nodules on the arms and forearm. On examination, a faint malar rash, Gottron’s sign, and erythema nodosum (EN) like lesions were noted on the right arm, forearm (**Figure 1A**), and thigh without overlying skin changes. Heliotrope rash, cutaneous ulcers and muscle weakness were absent. At this juncture, the EN-like lesions were believed to be due to an underlying panniculitis. A flare of DM was the prime differential, others being drug-induced, post-infectious, and granulomatous panniculitis. Although calcinosis and carcinomatous deposits presenting like this have been occasionally described in DM, these seemed unlikely. A skin biopsy confirmed lobar panniculitis with perivascular lymphocytic aggregates with vasculitis (**Figure 1B–D**). Tofacitinib was prescribed at 0.5 mg/kg GCs, to which she responded well with subsidence of skin lesions and normalisation of muscle enzymes 2 months later.

**Figure 1.**
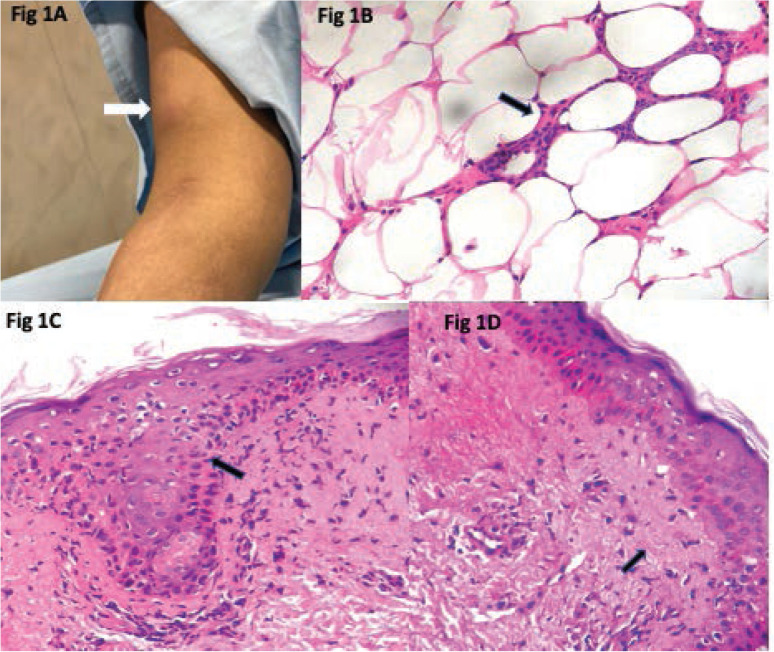
**(a)** Picture of the right arm and forearm depicting erythema nodosum like lesions. **(b)** Infiltration of inflammatory cells within the lipocyte lobules, surrounding the adipocytes (arrow). Inset show perivascular lymphocytic infiltrate; H&E stain; 400x. **(c)** Dermoepidermal inflammatory cells with focal infiltration of inflammatory cells in the epidermis (arrow); H&E stain; 400x. **(d)** Dermis shows mild peri-adnexal and perivascular lymphoplasmacytic infiltrate with foci of lymphocytic vasculitis (arrow).H&E stain; 400x.

Our case was unique in panniculitis being the transformed cutaneous manifestation of DM in a girl with muscle involvement at the outset, which progressed to a refractory amyopathic disease many years into the illness. Panniculitis, the inflammation of subcutaneous fat is rarely reported in DM. After the first published case in 1924, nearly 60 cases have been reported till date, most being in adults with female predominance.^1^ In most cases the panniculitis precedes or is concurrent with myositis, while in our case it appeared after four years of the disease. Typically, panniculitis presents as thickened and firm nodules or plaques with erythematous or pigmentation overlying skin with pain and tenderness as in our case. Post-infectious (bacterial, fungal, mycobacterial) and drug-induced EN are prime differentials.

The exact pathogenesis of panniculitis in myositis is unclear. On Histopathology, DM associated panniculitis shows predominantly lobular panniculitis along with lymphoplasmacytic infiltrate, and may rarely occur with vasculitis, as in our case. Depending on the aetiology, it may exhibit calcium, mucin, lipo-membranous changes, or rarely cancerous deposits.^3^ Panniculitis with vasculitis (as in our case) is even rarer. Previously around ten cases of DM associated panniculitis with vasculitis are reported. It may be a surrogate for more severe disease, and often requires aggressive management.^2^ Generally, panniculitis signify a more favourable prognosis than calcinosis.^3^ Complications like extreme pain, lipoatrophy and calcification may lead to high morbidity. Previous studies suggest successful management of EN with methotrexate, though panniculitis has reportedly occurred in certain cases while on methotrexate.^4^ MMF, CYC, and IvIg have been occasionally used for management.^5,6^ Our patient was unique in being refractory to numerous immunosuppressants at the outset, requiring the usage of JAKinibs for management of panniculitis. Although interferon signatures have not been studied in DM associated panniculitis, we expect the pathway to be operative and hope JAKinibs to manage the condition successfully.

## Figures and Tables

**Supplementary Table 1.** Baseline investigations at presentation.

**Investigation**	**Reference value (conventional units)**	**Value**
**Hemogram**		
Hemoglobin (gm/dl)	13–17	13.9
White blood count (per mm^3^)	4400–11000	14400
Platelet count (lakhs per mm^3^)	1.50–4.00	90000
**Muscle enzymes**		
Creatine phosphokinase (u/l)	20–192	303
Lactate dehydrogenase (u/l)	100–243	701
Alanine aminotransferase (u/l)	8–40	53
Aspartate aminotransferase (u/l)	8–40	85
**Autoantibodies**		
Myositis specific antibody	Negative	Negative
Anti-Nuclear antibody	Negative	2+ Homogenous
dsDNA antibody (IU/dl)	<10	42.2
ENA	Negative	SS-A/Ro 60 and 52 kd
**Acute phase reactants**		
ESR (mm/hr)	0–10	135
C reactive protein (mg/dl)	0–0.6	1.68
**Others**		
Creatinine (mg/dl)	0.6–1.5	0.7
Alkaline phosphatase (u/l)	60–270	311
Skin Biopsy	Negative	Panniculitis

Abbreviations- dsDNA- double stranded deoxy ribo-nucleic acid ENA- extractable nuclear antigen ESR- erythrocyte sedimentation rate

## AVAILABILITY AND DATA SHARING

The data used during the current study is available from the corresponding author on reasonable request.
